# Fetal movement counting is associated with the reduction of delayed maternal reaction after perceiving decreased fetal movements: a prospective study

**DOI:** 10.1038/s41598-021-90240-4

**Published:** 2021-05-24

**Authors:** Shigeki Koshida, Shinsuke Tokoro, Daisuke Katsura, Shunichiro Tsuji, Takashi Murakami, Kentaro Takahashi

**Affiliations:** 1 Department of Perinatal Center, Shiga University of Medical Science, Tsukinowa-cho, Seta, Otsu, Shiga 520-2192 Japan; 2 Department of Obstetrics and Gynecology, Shiga University of Medical Science, Tsukinowa-cho, Seta, Otsu, Shiga 520-2192 , Japan

**Keywords:** Epidemiology, Outcomes research, Preventive medicine, Public health, Epidemiology, Health care, Medical research, Diseases, Reproductive disorders, Signs and symptoms, Reproductive signs and symptoms

## Abstract

Maternal perception of decreased fetal movement is associated with adverse perinatal outcomes. Although there have been several studies on interventions related to the fetal movements count, most focused on adverse perinatal outcomes, and little is known about the impact of the fetal movement count on maternal behavior after the perception of decreased fetal movement. We investigated the impact of the daily fetal movement count on maternal behavior after the perception of decreased fetal movement and on the stillbirth rate in this prospective population-based study. Pregnant women in Shiga prefecture of Japan were asked to count the time of 10 fetal movements from 34 weeks of gestation. We analyzed 101 stillbirths after the intervention compared to 121 stillbirths before the intervention. In multivariable analysis, maternal delayed visit to a health care provider after the perception of decreased fetal movement significantly reduced after the intervention (aOR 0.31, 95% CI 0.11–0.83). Our regional stillbirth rates in the pre-intervention and post-intervention periods were 3.06 and 2.70 per 1000 births, respectively. Informing pregnant women about the fetal movement count was associated with a reduction in delayed maternal reaction after the perception of decreased fetal movement, which might reduce stillbirths.

## Introduction

Maternal perception of fetal movement (FM) is generally used as a sign of fetal well-being^1^. A reduction of FM is a critical fetal sign. Decreased fetal movement (DFM) is associated with adverse perinatal outcomes, including fetal growth restriction and stillbirths^2–4^. Pregnant women with stillbirths often perceived DFM before the diagnosis of intra uterine fetal demise (IUFD)^5^. We previously demonstrated that 36% of cases of IUFD were diagnosed at the time of a maternal visit to a health care provider due to the perception of DFM^6^, and that majority of these visits were excessively delayed after their perception of DFM^7^. We hypothesized that educating pregnant women about the risk of DFM would reduce the number of cases with an excessively delayed reaction to DFM.

There have been several studies on interventions involving the fetal movement count (FMC) that were performed with the aim of improving perinatal outcomes^8^. A recent Cochrane review that included only randomized controlled trials (RCTs) did not provide sufficient evidence to support that the formal FMC was beneficial for stillbirth prevention^9^. As RCTs can underestimate the effects of large public health interventions, non-RCTs, including before-after studies might be more useful for assessing the impact in such settings^8^. While most of the studies on interventions involving the FMC evaluated adverse perinatal outcomes, such as stillbirths, few studies have investigated both maternal behavioral changes after the perception of DFM and stillbirth^10^. Following on from this RCT, it would be useful to evaluate the effect of FMC in practice, which may give a more generalizable view about the clinical impact of FMC.

We prospectively assessed the impact of the daily FMC on maternal behavior after the perception of DFM. Our investigation included all stillbirths in our region before-after the intervention. We also evaluated regional stillbirth rates.

## Results

**Figure 1:**
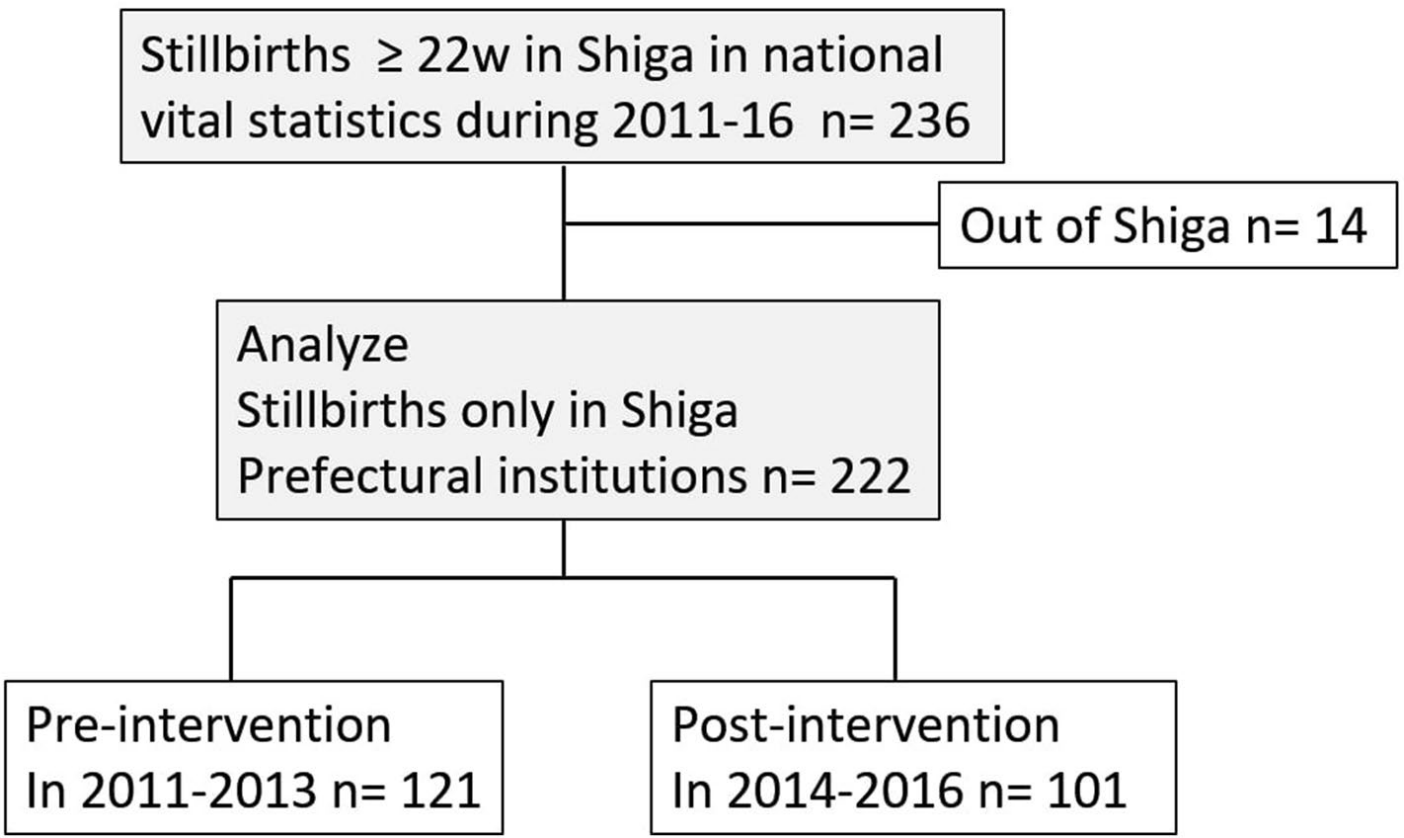
Overview of this study.

As Japanese vital statistics registers a stillbirth according to the certified residence, the statistics could include stillbirths delivered in another prefecture. To more accurately determine the effect of the intervention in our prefecture, we excluded 14 stillbirths that were delivered outside of Shiga Prefecture. Finally, we analyzed 222 stillbirths in this study period and divided them into two groups. A group of 121 stillbirths in the pre-intervention period (2011–2013) and a group of 101 stillbirths in the post intervention period (2014–2016) (Fig. 1).

### Characteristics

The characteristics of the study are described in Table 1. The proportion of women of ≥ 35 years of age at delivery was significantly higher in the post-intervention period. There were no significant differences in parity, gestational age, and maternally reported DFM between the two groups.

**Table 1:** Descriptive characteristics of total stillbirths in this study.

	Pre-intervention (2011–13) N = 121	Post-intervention (2014–16) N = 101	*P* value
Age (year)			0.036
< 35	90 (74)	69 (68)	
≥ 35	31 (26)	43 (43)	
Parity			0.42
Primiparous	60 (50)	54 (53)	
Multiparous	61 (50)	44 (44)	
GA			0.74
22–33 w	68 (56)	59 (58)	
≥ 34w	53 (44)	42 (42)	
Report of DFM			0.16
+	58 (48)	39 (39)	
−	63 (52)	62 (61)	
Stillbirths rate	3.06	2.70	0.35

*GA* gestational age, *DFM* decreased fetal movement. Stillbirths rate indicates per 1000births.

Data are n (%).

### Maternal behavior

Among stillbirths with maternal perception of DFM, the proportion of women with a delayed visit after the perception of DFM was significantly lower in the post-intervention group (46%) than in the pre-intervention group (67%) (Table 2). We excluded 15 stillbirths (pre-intervention, n = 9; post-intervention, n = 6) due to a lack of detailed information on the time after the perception of DFM. A multivariable logistic regression analysis showed that the rate of delayed maternal visits after the perception of DFM significantly decreased in the post-intervention group (adjusted OR, 031; 95% CI, 0.11–0.83; *p* = 0.02), after adjustment for maternal age and parity (Table 3).

**Table 2:** Among DFM population.

	Pre-intervention (2011–13) N = 58	Post-intervention (2014**–**16) N = 39	*P* value
Age (year)			0.26
< 35	46 (79)	27 (69)	
≥ 35	12 (21)	12 (31)	
Parity			0.77
Primiparous	33 (57)	21 (54)	
Multiparous	25 (43)	18 (46)	
GA			0.43
22–33 w	31 (53)	24 (62)	
≥ 34 w	27 (47)	15 (38)	
Visit time			0.016
< 24 h	10 (17)	15 (38)	
≥ 24 h	39 (67)	18 (46)	

*GA* gestational age, *DFM* decreased fetal movement. Visit time indicates the time to visit healthcare provider after maternal perception of DFM. The number of stillbirths without accurate visit time after perceiving DFM were excluded.

Data are n (%).

**Table 3:** The logistic regression analysis of delayed maternal visit of DFM.

	Pre-intervention n = 49	Post-intervention n = 33	Univariable		Multivariable	
			Crude OR (95% CI)	*p*	Adjusted OR (95% CI)	*p*
Delayed visit of DFM (≥ 24 h)	39 (80)	18 (55)	0.31 (0.12–0.82)	0.02	0.31 (0.11–0.83)	0.02

Adjusted for maternal age and parity. *OR* odds ratio, *CI* confidential interval, *DFM* decreased fetal movement.

Data are n (%).

### Impact on the stillbirth rate

The stillbirth rates in Shiga in pre-intervention and post-intervention periods were 3.06 and 2.70 per 1000 births, respectively; the reduction was not statistically significant (*p* = 0.35) (Table 1)^11^. Among the DFM population, the rates of stillbirths at ≥ 34 weeks of GA in the pre-intervention and post-intervention periods were 47% and 38%, respectively (*p* = 0.43) (Table 2).

## Discussion

In this prospective study analyzing 222 stillbirths in our region over a 6-year period, we revealed that informing pregnant women about the daily FMC was associated with a reduction in delayed maternal reaction after the perception of DFM. We also showed that the stillbirth rate in our region was non-significantly reduced in the post-intervention period.

First, we found that informing pregnant women about the daily FMC was associated with a reduction in the rate of delayed maternal reaction after the perception of DFM. The results of our study, in which only stillbirth cases were analyzed, were consistent with a previous study by Saastad et al.^10^ which mostly targeted cases with few stillbirths. While their study analyzed few cases with fetal demises, we found a similar association in our analysis, which was limited to stillbirth cases. We ascertained that FMC could affect maternal behavior after their perception of DFM. While the study of Saastad et al. revealed that a significant behavioral change in the reporting of DFM was only obtained in primiparous women^10^, our study showed a significant change in the maternal behavior of all pregnant women, including multiparous women. Pregnant women seem poorly informed about the significance of FM in general, and health care professionals have varying recommendations about normal fetal movements^12^. Maternal awareness of changes in FM according to the daily FMC might have contributed to a significant reduction in delayed maternal visits after the perceiving of DFM.

Next, we showed a non-significantly reduction in the stillbirth rates in our region in the post-intervention period. A meaningful reduction in the stillbirth rate in our region was observed; however, the result did not reach statistical significance due to the small sample size. The non-significant difference of stillbirth rates between pre-intervention and post-intervention groups is consistent with a recent systematic review of RCTs by Bellussi et al.^13^. They suggested that instructing pregnant women on FMC was associated with a non-significant 8% reduction^14,15^, which was similar to the decrease in our study (12%). Another recent systematic review by Winje et al. also showed a non-significant reduction in stillbirths following an intervention to enhance maternal awareness of DFM with or without FMC^8^. Although recent systematic reviews did not show that maternal FMC intervention was associated with a significant reduction in the rate of stillbirth^8,9,13^, this does not necessarily indicate that maternal education on FM is ineffective in relation to the prevention of adverse perinatal outcomes, for the following reasons. First, other perinatal outcomes such as cases successfully rescued by interventions were not assessed. Perinatal clinicians often experience cases that are rescued after an unscheduled maternal visit due to the perception of DFM^15^. Although it is difficult to count such rescued cases, they should be evaluated as cases in which the intervention was associated with a good perinatal outcome. Second, pregnant women could naturally perceive their FM, even without a formal daily FMC. DFM is the most common reason for an unscheduled visit^12^ and DFM was perceived in 44% of the stillbirth in the current study. The provision of pregnant women with accurate information on FM and the earlier report of DFM is beneficial for reducing maternal anxiety in relation to FM. Third, interventions associated with the FMC do not increase maternal concern^16–18^. Education on FM is acceptable to pregnant women. Thus, in order to prevent stillbirths, pregnant women should be informed about the importance of both awareness in relation to fetal movement and an earlier reaction after the perception of DFM.

The present study was associated with some limitations. First, we could not determine the accurate number of interventions. We introduced FMC to pregnant women through obstetric institutions in our region. Several institutions did not participate in the current study and two primary clinics out of 30 ones had introduced FMC before the study intervention. Furthermore, our study has a participant bias including only women having a stillbirth. We did not include women with live births, because it was extremely difficult to obtain data on all women who gave births at as many as 30 obstetric facilities in our region. Consequently, the sample size in our study was much smaller than in previous studies on the association between maternal perception of DFM and adverse perinatal outcomes. However, it is possible to suggest that the maternal behavior significantly changed after the regional intervention. Next, the change in the stillbirth rate might have been affected by factors other than the intervention. The stillbirth rate in Japan has been decreasing for several decades. The improvement of perinatal outcomes might have been influenced by several factors, including the development of perinatal care and assistance in allocation for perinatal medical systems. Although it might be difficult to assess the effects of the intervention on the stillbirth rate as a major outcome, we consider that informing pregnant women about FM could be beneficial for the improvement of adverse perinatal outcomes. Finally, we did not indicate the association between maternal perception of DFM and the cause of stillbirth. Several studies demonstrated the association between DFM and placental pathology^19,20^. As approximately half of the primary causes of stillbirths in our study could not be identified, we did not evaluate their association.

In conclusion, the present study revealed that informing pregnant women about the fetal movement count was associated with a reduction in delayed maternal reaction after the perception of DFM. The non-significant decrease in the stillbirth rate after the intervention may contribute to the further study on the impact of maternal education on FM on adverse perinatal outcomes.

## Methods

### Study design and ethical statement

This was a population-based study in Shiga prefecture of Japan and it was prospectively planned to analyze the effect of rolling out the FMC. This study was approved by the Institutional Review Board of Shiga University of Medical Science on March 25, 2014 (Approval No. 25-211). Informed consent was obtained from all participants for FM counting, and we implemented an opt-out method by posting on the website to obtain the consent of participants with stillbirth on this study. This study was performed in accordance with the Declaration of Helsinki.

### Intervention

**Figure 2:**
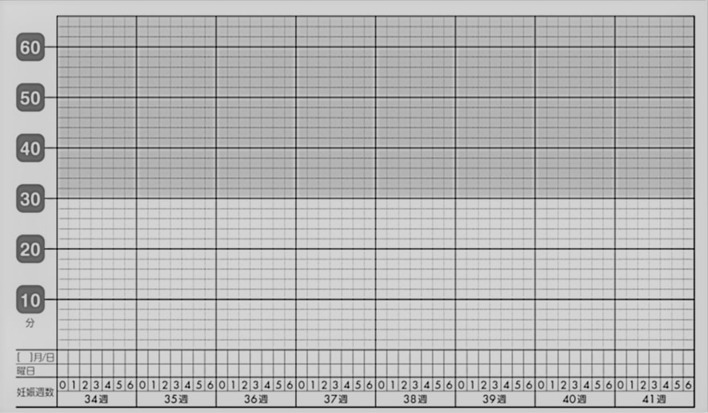
Fetal movement counting chart. Pregnant women were asked to record the time taken to perceive 10 fetal movements from 34 weeks of gestation, and also asked to report if it took more than 30 min to perceive 10 fetal movements.

This was another analysis of our previous study investigating FM frequency, which was conducted in Shiga^21^. We made an original FM counting chart and provided it to each pregnant woman through an obstetric caregiver in our region from May 2014 to April 2017 (Fig. 2). Pregnant women were asked to record the time taken to perceive 10 FMs by the modified 'count to 10' method on the chart once per day at any time from 34 weeks of gestation until their delivery. They were also asked to report to their caregiver if it took more than 30 min for them to perceive 10 FMs.

### Data collection

The annual number of births in Shiga is approximately 13,000. Two-thirds of the deliveries are performed in 30 primary obstetrics clinics; the remainder are performed at 6 general hospitals and 4 perinatal centers. We inspected all stillbirth certificates for stillbirth deliveries at ≥ 22 weeks of gestational age (GA) in Shiga with permission of the Japanese Ministry of Health, Labor, and Welfare. Based on the information, we sent a questionnaire to each obstetrician who had submitted a stillbirth certificate. The questionnaire included perinatal maternal characteristics and the information about the maternal perception of DFM and the time from the perception of DFM to visiting an obstetrics facility. We obtained detailed data on stillbirth cases from the returned questionnaires. We defined DFM as based on the description of perceived decreased fetal movement or a loss of fetal movements on the maternal report.

### Outcome measures

The primary outcome measure was the rate of stillbirths with an excessive delayed maternal reaction (≥ 24 h) after the perception of DFM among stillbirths with the perception of DFM before and after intervention. The secondary outcome measure was the rate of stillbirths in our region in each period.

### Statistical analyses

All statistical analyses were performed using the IBM SPSS software program, (ver.22 IBM Japan, Tokyo, Japan). Chi-squared tests were used to estimate differences between rates of categorical variables. To determine the change of the maternal reaction after the perception of DFM between the pre- and post-intervention periods, we performed multivariable logistic regression analyses of the DFM population. Adjusted odds ratios (ORs) with 95% confidence intervals (CIs) were reported. *P* values of < 0.05 were considered to indicate statistical significance.
